# Effectiveness of Active-Online, an Individually Tailored Physical Activity Intervention, in a Real-Life Setting: Randomized Controlled Trial

**DOI:** 10.2196/jmir.1179

**Published:** 2009-07-28

**Authors:** Miriam Wanner, Eva Martin-Diener, Charlotte Braun-Fahrländer, Georg Bauer, Brian W Martin

**Affiliations:** ^4^Center for Occupational and Organizational Sciences ETH ZurichZurichSwitzerland; ^3^Institute of Social and Preventive MedicineUniversity of ZurichZurichSwitzerland; ^2^Institute of Social and Preventive MedicineUniversity of BaselBaselSwitzerland; ^1^Swiss Federal Institute of Sport MagglingenMagglingenSwitzerland

**Keywords:** Effectiveness, tailored intervention, adults, Internet, physical activity

## Abstract

**Background:**

Effective interventions are needed to reduce the chronic disease epidemic. The Internet has the potential to provide large populations with individual advice at relatively low cost.

**Objective:**

The focus of the study was the Web-based tailored physical activity intervention Active-online. The main research questions were (1) How effective is Active-online, compared to a nontailored website, in increasing self-reported and objectively measured physical activity levels in the general population when delivered in a real-life setting? (2) Do respondents recruited for the randomized study differ from spontaneous users of Active-online, and how does effectiveness differ between these groups? (3) What is the impact of frequency and duration of use of Active-online on changes in physical activity behavior?

**Methods:**

Volunteers recruited via different media channels completed a Web-based baseline survey and were randomized to Active-online (intervention group) or a nontailored website (control group). In addition, spontaneous users were recruited directly from the Active-online website. In a subgroup of participants, physical activity was measured objectively using accelerometers. Follow-up assessments took place 6 weeks (FU1), 6 months (FU2), and 13 months (FU3) after baseline.

**Results:**

A total of 1531 respondents completed the baseline questionnaire (intervention group n = 681, control group n = 688, spontaneous users n = 162); 133 individuals had valid accelerometer data at baseline. Mean age of the total sample was 43.7 years, and 1146 (74.9%) were women. Mixed linear models (adjusted for sex, age, BMI category, and stage of change) showed a significant increase in self-reported mean minutes spent in moderate- and vigorous-intensity activity from baseline to FU1 (coefficient = 0.14, P = .001) and to FU3 (coefficient = 0.19, P < .001) in all participants with no significant differences between groups. A significant increase in the proportion of individuals meeting the HEPA recommendations (self-reported) was observed in all participants between baseline and FU3 (OR = 1.47, P = .03), with a higher increase in spontaneous users compared to the randomized groups (interaction between FU3 and spontaneous users, OR = 2.95, P = .02). There were no increases in physical activity over time in any group for objectively measured physical activity. A significant relation was found between time spent on the tailored intervention and changes in self-reported physical activity between baseline and FU3 (coefficient = 1.13, P = .03, intervention group and spontaneous users combined). However, this association was no longer significant when adjusting for stage of change.

**Conclusions:**

In a real-life setting, Active-online was not more effective than a nontailored website in increasing physical activity levels in volunteers from the general population. Further research may investigate ways of integrating Web-based physical activity interventions in a wider context, for example, primary care or workplace health promotion.

## Introduction

To reduce the burden of chronic disease and premature death due to an inactive lifestyle [1-3], interventions are needed that are effective in enhancing physical activity levels in the general population. In Switzerland, the health-enhancing physical activity (HEPA) recommendations advocate at least 30 minutes of moderate activity on most, preferably all, days of the week or at least 20 minutes of vigorous activity on three or more days of the week [4]. However, only 36% of the adult population in Switzerland meets either of these recommendations [5]. Thus, effective interventions reaching large numbers are required.

Computer-tailored interventions simulate a personal counseling situation by providing individual feedback based on the behavior, motivation, and attitudes of the user [6]. Tailored interventions of the first generation (using print materials for assessment and dissemination) have been effective in inducing behavior changes for smoking [7,8], nutrition [9,10], and physical activity [11-15]. Second-generation interventions use the advantages of the Internet—interactivity, availability at any time from any place, and immediate display of feedback—to potentially reach large populations at relatively low cost.

To date, studies investigating the effectiveness of second-generation Web-based tailored physical activity interventions have either been carried out in small confined populations [16-18], have not used truly tailored information but materials targeted to the stages of change [19,20], have looked at only short-term effects [21], or have been carried out in optimized and controlled settings such as computer labs [22]. Results from these studies were mixed [23,24]. Interventions shown to be effective in controlled settings may still be ineffective if delivered in an uncontrolled, real-life setting. The potential public health impact of Web-based computer-tailored interventions can only be estimated if their effectiveness is tested under real-life conditions. Thus, studies evaluating online physical activity interventions in real-life settings are now needed. To our knowledge, there is only one individually tailored Internet-based intervention targeting physical activity that has been evaluated in two samples of the general population in a real-life setting, showing mixed results [25,26].

Intensity of intervention use may be associated with induced physical activity changes [27]. However, little is known about the impact of frequency and duration of intervention use on the effectiveness of a Web-based tailored physical activity intervention. This may be an important issue for the interpretation of results from real-life effectiveness studies.

The focus of the present study was a Web-based tailored physical activity intervention that is freely accessible on the Internet [28]. Active-online was tested for its acceptability and feasibility before the final version went online in 2003 [29]. The main research questions were (1) How effective is Active-online, compared to a nontailored website with general information on physical activity and health, in increasing self-reported and objectively measured physical activity levels in the general population when delivered in a real-life setting? (2) Do respondents recruited for the randomized study differ from spontaneous users of Active-online, and how does effectiveness differ between these groups? (3) What is the impact of frequency and duration of use of Active-online on changes in physical activity behavior?

## Methods

### Study Design, Setting, and Participants

Participants for this Web-based study were recruited by advertisements in newspapers, in magazines, and on the Internet. They were invited to take part in a physical activity study and were given the link to the study website (with a domain name different from the one of the intervention). At the same time, spontaneous users were recruited directly from the Active-online website by redirecting them to the study website if they chose to participate in the study. The study was carried out in German, and recruitment lasted from May 1 to August 2, 2006. Based on sample size calculations assuming an increase in meeting the HEPA recommendations of 30% in the intervention group and 20% in the control group (alpha = .05, power = 0.8), 250 participants were required per group. Assuming a realistic loss-to-follow-up in a Web-based survey without face-to-face contact of about 50% over 1 year [30], this number doubled to 500 participants per group.

Interested individuals could access the study website from any computer with Internet access. Information regarding the study and all study questionnaires were provided there. Individuals completing the baseline questionnaire and leaving their email address were registered. Media-recruited participants were randomly allocated to either the intervention group (IG) or the control group (CG) and were forwarded to Active-online or the nontailored website, respectively. Spontaneous users (SU) were included as a separate study group but followed the same study protocol as the IG.

Respondents could volunteer to take part in accelerometer measurements via an additional Web page that they were routed to after the baseline questionnaire, depending on the availability of accelerometers. Volunteers were not forwarded directly to the intervention websites but were sent an accelerometer to obtain baseline measurements and had to return a separate written consent form. Only after the accelerometer was returned was an email sent out with a link to Active-online or the nontailored website.

Randomization was carried out using random numbers provided by the University of Geneva’s online service [31] based on a physical quantum random number generator. Participants were not aware of the group they were randomized to. The study website with all the study questionnaires was kept strictly separate from Active-online, using two different domains, in order to minimize the chance of controls getting involved with Active-online.

**Figure 1:**
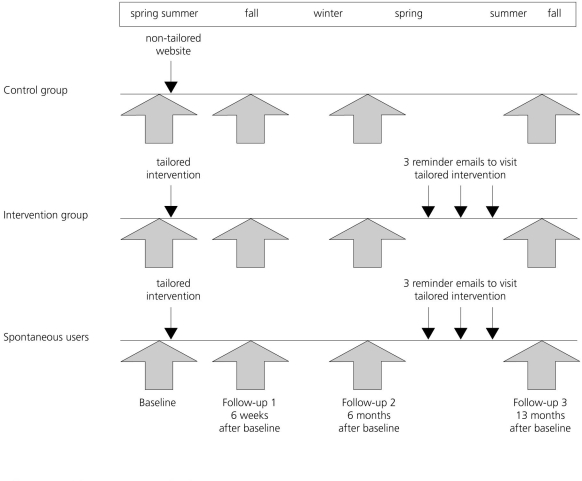
Study procedure for each group

Email addresses were used to identify and contact participants at follow-up. All participants were followed up 6 weeks (FU1), 6 months (FU2), and 13 months (FU3) after the baseline assessment, receiving a maximum of three email invitations each time with a personal link referring them back to the study website. Those volunteers having participated in the accelerometer measures at baseline were asked to repeat accelerometer measures at each follow-up in addition to the online questionnaires. Individuals in the IG and SU additionally received three reminder emails with a personal link to Active-online between FU2 and FU3 at 9, 10, and 11 months after the baseline assessment, encouraging them to revisit Active-online. The study procedure is depicted in Figure 1 according to group. The study used an automated design with emails automatically timed to each participant’s starting date. There were no face-to-face contacts. The study design had been tested in a feasibility study [32]. As incentives, two city bikes were being raffled among the participants who completed the study. The study was approved by the ethics commission of the canton of Berne, Switzerland. The trial was not registered as the funding agency (Swiss Federal Council of Sports ESK) did not request a trial registration and we were not aware when the study started in 2006 that web-based trials should be registered. In lieu of trial registration, we append the original application for funding, containing the protocol (Multimedia Appendix 1).

### Tailored Intervention and Standard Website

**Figure 2:**
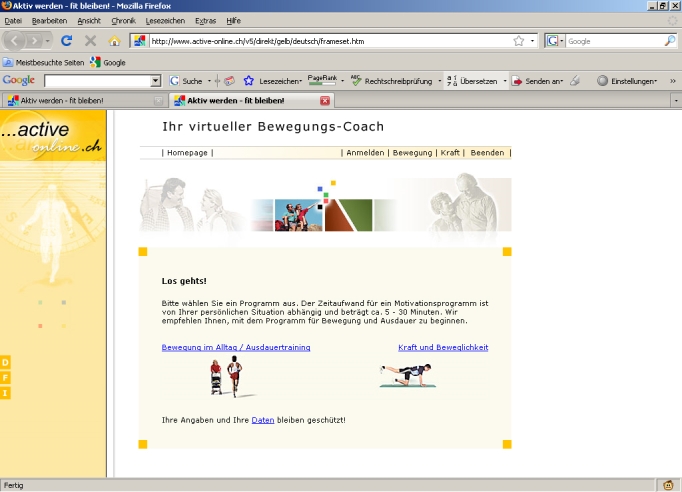
Screenshot of the tailored intervention

Active-online is an interactive, individually tailored physical activity program targeting individuals aged 30 to 60 years. It has been freely available on the Internet since 2003. The aim of the program is to increase physical activity levels in users by offering individually tailored counseling and motivational feedback. The program was developed in German by an interdisciplinary team of experts in public health, sport sciences, psychology, design, and computer science, and then translated and culturally adapted for French and Italian audiences. The theoretical framework of the program is the transtheoretical model of behavior change [33]. Visitors may choose one of two tailored modules, either on everyday activities and endurance training or on strength and flexibility training. Figure 2 shows a screenshot of the Active-online page where one of the two modules can be selected. The first module offers a maximum of four tailored feedbacks using questionnaires on stages of change, decisional balance, processes of change, and self-efficacy. Stages of change are assessed according to a seven-stage concept focusing on current behavior (moderate- and vigorous-intensity activity) as well as on intention to change [34]. The decisional balance and self-efficacy scales are based on instruments of Basler et al [35], and the processes of change scale on instruments of Marcus et al [36], and Nigg and Riebe [37]. Depending on their current stage of change, visitors are guided trough one, three, or four sections of the module. More information is available in Martin-Diener et al [34]. The module on strength and flexibility training offers a maximum of two tailored feedbacks based on questionnaires assessing five stages of change as well as attitudes and knowledge regarding strength training. The feedback for flexibility training is based on current behavior. Based on answers to these “diagnostic” questionnaires, short text segments are selected from the feedback library, compiled into unique individual feedback, and displayed immediately on screen. Feedback reports are available in a printer-friendly format. Additional support tools, such as strength and stretching exercise sheets, and organizational and motivational download forms, are provided.

Users may visit Active-online without registering, or they may register with their email address to obtain a password. Registered users have the possibility of following changes in their physical activity behavior when revisiting the website. They also receive reminder emails encouraging them to revisit Active-online.

**Figure 3:**
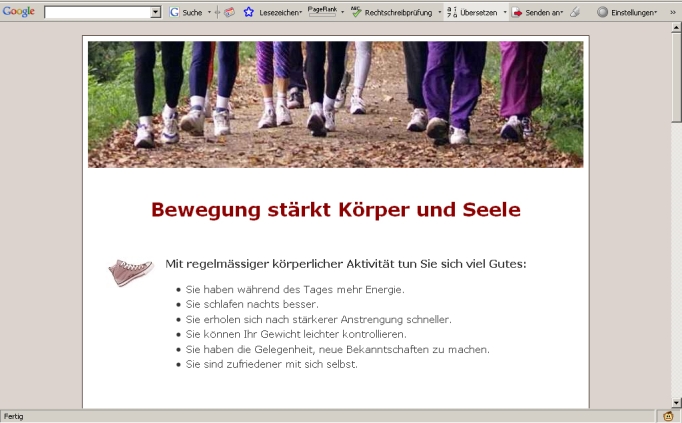
Screenshot of the standard website for the CG

Participants in the CG were forwarded to a nontailored website with general information on physical activity and health with no additional reminder emails. This was a static website with some tips on how to include more physical activity in daily life and some information regarding positive health effects of physical activity. Figure 3 shows a screenshot of the nontailored website.

### Measures

The online baseline questionnaire included questions on demographics, physical activity behavior, stage of change, self-efficacy, and general and mental health. Data are only presented for physical activity. Physical activity was assessed using a short questionnaire with four items on frequency and duration of moderate- and vigorous-intensity activity that is used in the official monitoring of physical activity in the Swiss population [38]. These questions allow the calculation of total minutes of moderate- and vigorous-intensity activity per week (total reported activity time) and the classification of participants according to the HEPA recommendations as outcome measures. Reported times exceeding 8 hours per day or 40 hours per week of moderate-intensity activity and 5 hours per day or 17.5 hours per week of vigorous-intensity activity were set to missing. Truncating these high values instead of setting them to missing did not change the results. Demographic variables included age, gender, living situation, highest education, nationality, smoking status, height, and weight. Body mass index (BMI) was calculated as weight (in kg) divided by height (in meters squared) and categorized as < 18.5, 18.5 to < 25, 25 to < 30, and ≥ 30 kg/m^2^. The same questionnaire (except demographic variables) was used in the follow-up assessments.

Accelerometers (Actigraph models AM7164 and GT1M, formerly Computer Science and Applications, now Manufacturing Technology Inc, Fort Walton Beach, FL, USA) were used for objective physical activity assessment. The accelerometers have been validated in earlier studies [39,40]. Participants were asked to wear the accelerometer on their right hip during waking hours for a 7-day period at baseline and each follow-up. A minimum of 4 days with at least 10 hours per day of data recording, including one weekend day, were required to be included in the analysis. The data collected by the accelerometers are a series of counts integrating vertical acceleration over a specified time interval (epoch time). Epoch time was set to 1 minute. Cut-off points developed by Swartz et al were used to classify light (≤ 573 counts/minute), moderate (574-4944 counts/minute), and vigorous activities (≥ 4945 counts/minute) [41]. These cut-offs were chosen because they were derived using a wide range of lifestyle activities and may prove applicable for predicting time spent in different intensity categories during free-living activities [42]. Mean counts per minute over the recording period and minutes of moderate and vigorous activity per week according to accelerometer data (total accelerometry activity time, only bouts of at least 10 minutes) were calculated.

Data regarding the use of Active-online for the IG and SU were obtained from the Active-online user database. Each visit to the website was recorded, including start date and time, end date and time, number of pages viewed, etc. Participants were provided with a password to re-enter Active-online in order to track their repeated visits. Use of the nontailored website in the CG was not measured.

### Statistical Analyses

Minutes of physical activity were positively skewed and were log-transformed for analysis. Chi-square tests for categorical variables and t-tests for continuous variables were used to compare responders and nonresponders and to compare differences between IG and CG and between IG and SU at baseline. In a preliminary analysis, paired t-tests and McNemar tests were applied, respectively, to compare changes in total activity time and changes in the proportion meeting the HEPA recommendations between baseline and each FU and for each group separately. Mixed logistic and mixed linear models were used to simultaneously analyze the effects of time and group allocation on the proportion meeting the HEPA recommendations and on total activity time, respectively, including gender, age, BMI category, and stage of change at baseline as covariates in the adjusted model. Stage of change was included to account for baseline motivation to change. The inclusion of time-group interaction terms in mixed models allows identification of potential differences in changes between groups at any time point. Changes in total reported activity time were analyzed for all participants and separately for participants meeting and not meeting the HEPA recommendations at baseline, because the latter are those most in need of effective interventions to increase their physical activity behavior. Participants were analyzed as randomized.

The impact of the use of Active-online on changes in physical activity behavior in the IG and SU was analyzed with a linear regression model including the difference in total reported activity time between baseline and FU3 as the dependent variable and the minutes spent in the tailored intervention as the independent variable, including gender, age, BMI category, and stage of change at baseline as covariates in the adjusted model. STATA 9.2 (STATACorp LP, College Station, TX, USA) was used for all analyses.

## Results

### Participants

**Figure 4:**
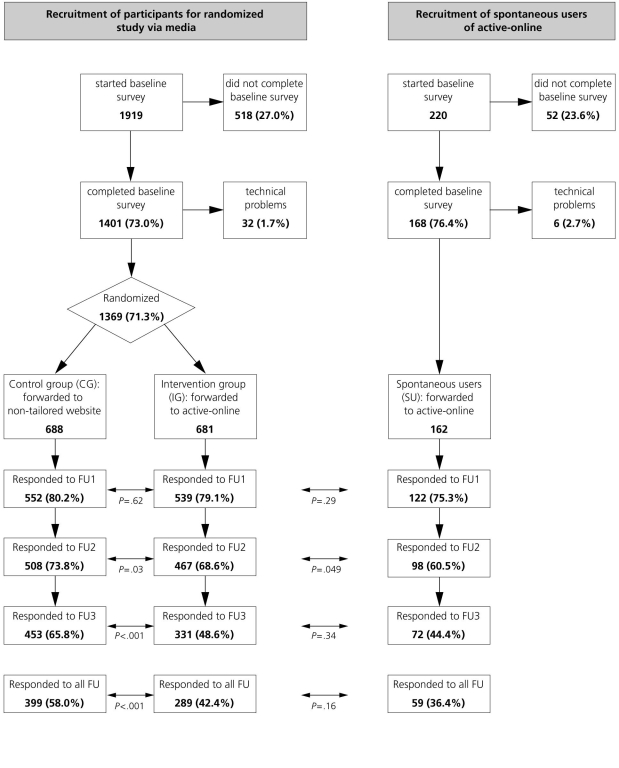
Participant flow: recruitment channels, randomization, baseline, and follow-up assessments

In total, 1919 respondents recruited via different media channels and 220 respondents recruited via the Active-online website started the baseline survey on our study website; 1401 and 168, respectively, finished the survey and were registered as participants (Figure 4). We excluded 38 respondents due to technical problems; 1369 were randomized into the IG (n = 681) or the CG (n = 688), and 162 were registered as SU (7.4% of all visits recorded in the Active-online user database during the recruitment period). The response is shown in Figure 4 according to group allocation and follow-up. No difference in response was seen at FU1. At FU2, response was significantly lower in the IG compared to CG, and in SU compared to IG. At FU3, response was significantly lower in both the IG and SU compared to CG, with no significant difference between IG and SU. Depending on availability of accelerometers, 326 participants (21.3%) had the choice to wear an accelerometer. Of those, 144 (44.2%) agreed to take part in the objective measures, corresponding to 9.4% of the total sample.

Table 1 displays the baseline characteristics of the total sample and of each group separately. There were no significant differences in demographic variables between the randomized groups (IG and CG). However, compared to the IG, SU were significantly younger (38.8 versus 44.2 years, P < .001), more likely to be smokers (23.5% versus 12.8%, P < .001), and less likely to be living with children (38.9% versus 53.5%, P < .001).

**Table 1:** Characteristics of participants at baseline according to group^a^

Self-Reported Measures	Total (n = 1531)	CG (n = 688)	IG (n = 681)	P(IG-CG)	SU (n = 162)	P(IG-SU)
**Demographic variables**						
Female (%)	74.9	75.9	74.7	.63	71.0	.33
Age, years	43.7 ± 13.1	44.2 ± 12.8	44.2 ± 13.3	.99	38.8 ± 13.0	< .001
Age groups (%)				.32		< .001
< 30 years	16.2	13.8	15.3		30.3	
30-60 years	72.9	75.6	72.1		64.8	
> 60 years	10.9	10.6	12.6		4.9	
Living with a partner (%)	70.0	70.4	70.8	.86	65.4	.18
Living with children (%)	53.2	56.3	53.5	.30	38.9	.001
Swiss nationality (%)	87.3	86.2	88.6	.19	86.4	.45
University degree (%)	24.9	25.2	24.1	.65	27.2	.41
**Health-related variables**						
Smokers (%)	13.1	10.9	12.8	.28	23.5	.001
BMI, kg/m^2^	24.6 ± 4.6	24.5 ± 4.5	24.8 ± 4.6	.38	24.5 ± 4.6	.57
Overweight and obese (%)	39.3	38.3	41.1	.30	36.4	.28
**Physical activity-related variables**						
Meeting HEPA recommendations (%)	40.8	40.4	40.9	.84	42.2	.75
Total reported activity time, minutes/week	277 ± 253	276 ± 256	276 ± 258	.99	283 ± 222	.76

Objective Measures	Total (n = 133)	CG (n = 52)	IG (n = 62)	P(IG-CG)	SU (n = 19)	P(IG-SU)
**Objective physical activity**						
Mean counts per minute	451 ± 186	450 ± 176	457 ± 196	.85	436 ± 193	.69
Total accelerometry activity time, minutes/week	377 ± 214	383 ± 211	383 ± 227	.99	341 ± 183	.47

^a^Values are mean ± SD unless otherwise noted.

There were significant differences in some variables between participants who responded to each follow-up (responders) and those who did not respond to at least one follow-up (nonresponders). Nonresponders were slightly younger, less likely to be Swiss, more likely to be smokers at baseline, more likely to be overweight or obese, and less likely to meet the HEPA recommendations at baseline. The subgroup of participants with accelerometers (n = 144) were slightly older, more likely to live with children, and more likely to be overweight or obese than those not participating in the accelerometer part of the study.

### Self-Reported Physical Activity

**Figure 5:**
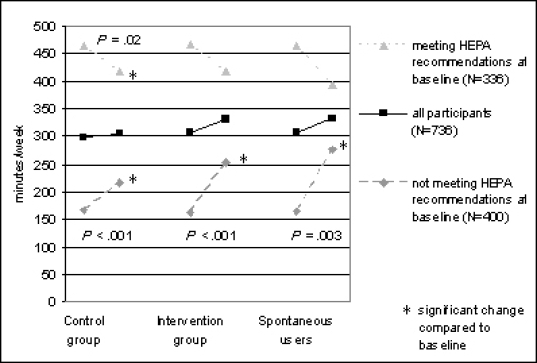
Changes in total reported activity time (minutes/week) between baseline and FU3 according to group, for all participants with complete data (n = 736) and separately for those meeting (n = 336) and not meeting (n = 400) the HEPA recommendations at baseline

When including those participants with complete data for all four time points (n = 736), significant increases in the proportion of participants meeting the HEPA recommendations were observed in SU between baseline and FU1 (P = .045) and FU3 (P = .002). Nonsignificant increases between baseline and FU3 were seen in the IG and CG. Changes in total reported activity time per week between baseline and FU3 are depicted in Figure 5 according to group, for all participants with complete data (n = 736) and separately for those individuals meeting (n = 336) and not meeting (n = 400) the HEPA recommendations at baseline. When including only those participants who did not meet the HEPA recommendations at baseline, total reported activity time increased significantly in all groups. The increases observed in these insufficiently active individuals exceeded the increase observed in all participants; thus, a decrease in total reported activity time was found in those individuals meeting the HEPA recommendations at baseline. The decrease was significant in the CG.

Table 2 shows the percent changes in the proportion meeting the HEPA recommendations and changes in total reported activity time between baseline and each follow-up according to group, including those participants who responded to the specific follow-up. A significant increase in individuals meeting the HEPA recommendations was observed in SU between baseline and FU3. Total reported activity time was generally lower at FU2; however, there were no significant changes at any follow-up or in any group.

**Table 2:** Percent changes in self-reported physical activity between baseline and each follow-up according to group^a^

	Baseline to FU1	*P*	Baseline to FU2	*P*	Baseline to FU3	*P*
**Meeting HEPA recommendations**						
CG	+2.2%	.27	−0.8%	.71	+3.8%	.12
IG	+2.3%	.27	−1.7%	.45	+4.0%	.21
SU	+7.4%	.11	−2.0%	.69	+18.3%	.005
**Total reported activity time (minutes/week)**						
CG	+4.8%	.13	−3.7%	.27	+4.5%	.25
IG	+4.7%	.14	−2.6%	.52	+3.4%	.51
SU	+9.6%	.11	+4.9%	.48	+15.4%	.19

^a^Results are based on participants with complete data at two time points (see Figure 4 for number of participants).

Table 3 shows the results from mixed logistic and mixed linear models for all participants, evaluating simultaneously the effect of time and group allocation. There were no differences between groups regarding the HEPA recommendations; however, a borderline significant increase in total reported activity time was found for SU compared to the CG. Irrespective of group allocation, participants were significantly more likely to meet the HEPA recommendations at FU3 compared to baseline, and a significant increase in total reported activity time was observed between baseline and FU1 as well as between baseline and FU3. There was a significant interaction between FU3 and SU in both the unadjusted (OR = 2.83, P = .03) and adjusted logistic model (OR = 2.95, P = .02), indicating that the proportion meeting the HEPA recommendations was significantly higher in SU compared to the randomized groups at FU3. There were no interactions between time of follow-up and group allocation in the mixed linear model, indicating that there were no differences in total reported activity time between groups at any follow-up.

**Table 3:** Time and group parameters for changes in physical activity, based on mixed logistic and mixed linear models^a^

	Meeting HEPA Recommendations				Total Reported Activity Time (minutes/week)			
	Unadjusted		Adjusted		Unadjusted		Adjusted	
	OR	95% CI	OR	95% CI	Coeff	95% CI	Coeff	95% CI
**Group**								
IG	1.04	0.68-1.57	1.02	0.72-1.45	0.02	−0.10, 0.15	0.02	−0.08, 0.13
SU	1.15	0.59-2.24	1.08	0.62-1.89	0.16	−0.04, 0.35	0.17	0.000-0.35
**Time**								
FU1 (6 weeks)	1.34	0.96-1.85	1.30	0.93-1.82	0.15	0.06-0.23	0.14	0.05-0.22
FU2 (6 months)	1.04	0.75-1.46	1.01	0.71-1.42	0.02	−0.06, 0.11	0.02	−0.07, 0.10
FU3 (13 months)	1.49	1.05-2.11	1.47	1.03-2.09	0.19	0.10-0.28	0.19	0.10-0.28

^a^Basic unit is the CG at baseline. Adjusted models include gender, age, BMI category, and stage of change at baseline.

### Objectively Measured Physical Activity

At baseline, 144 individuals (56 in CG, 68 in IG, and 20 in SU) wore an accelerometer, resulting in valid data for 133 individuals (92.4%). Valid accelerometer data were available for 117 individuals (88.0% of those with valid data at baseline) at FU1, for 114 individuals (85.7%) at FU2, and for 105 individuals (78.9%) at FU3; 93 participants (69.9%) had complete accelerometer data. There were no differences between groups.

Table 4 shows the percent changes in counts per minute and in total accelerometry activity time between baseline and each follow-up for each group separately. There were no significant changes observed in the IG. In the CG, activity levels decreased significantly between baseline and FU2 as well as between baseline and FU3. In SU, activity levels decreased significantly between baseline and FU2. Mixed linear models did not show any significant effects for time and group and no interaction effects.

**Table 4:** Percent changes in objective physical activity between baseline and each follow-up according to group^a^

	Baseline to FU1	*P*	Baseline to FU2	*P*	Baseline to FU3	*P*
**Counts per minute**						
CG	−4.8%	.19	−11.6%	.004	−8.1%	.03
IG	+2.5%	.52	−8.0%	.06	−1.2%	.83
SU	+1.5%	.83	−16.2%	.04	−2.1%	.81
**Total accelerometry activity time (minutes/week)**						
CG	−6.2%	.29	−17.5%	< .001	−10.3%	.03
IG	−1.5%	.82	−5.4%	.29	−1.2%	.87
SU	−5.1%	.72	−29.1%	.045	−16.0%	.16

^a^Results are based on participants with complete data at two time points.

### Frequency and Duration of Use of Active-Online

In total, 2112 visits of IG and SU study participants (n = 843) were counted on Active-online, with a mean number of 2.5 (± 1.6) visits per person. The number of visits was described by a positively skewed distribution representing 50% with two or less visits on Active-online during the study period between baseline and FU3. On average, 46 pages were viewed per person, with a median of 31 pages.

In 1226 of all visits (58.0%), one of the two tailored modules was started. These 1226 visits can be attributed to 628 individuals (74.5% of all participants in IG and SU). The mean number of visits within a tailored module for these individuals was 1.9 (± 1.2). The mean and median time spent in the modules for participants who started a tailored module was 12 minutes and 9 minutes per visit, respectively, and 23 minutes and 15 minutes during the whole study period, respectively.

In 962 of all visits (45.5%), at least one tailored feedback was obtained in the module on everyday activities and endurance training, and in 460 of all visits (21.8%), at least one tailored feedback was obtained in the module on strength and flexibility training. There was no difference in the use of Active-online between the IG and SU.

In the CG, 62 of 453 participants responding to FU3 (13.7%) stated that they had heard about Active-online and had used it at least once during the preceding year.

Linear regression showed a weak but significant relation between total minutes spent within one of the tailored modules (IG participants and SU combined) and changes in total reported activity time between baseline and FU3 in the unadjusted model (coefficient = 1.13, 95% CI 0.09 - 2.17, P = .03), and a borderline significant relation in the model adjusted for age, gender, and BMI category (coefficient = 1.07, 95% CI 0.004 - 2.13, P = .049). When adding stage of change to the model, the relation was attenuated and no longer significant (coefficient = 0.58, 95% CI −0.43 to 1.59, P = .26), indicating that stage of change was associated with both changes in total reported activity time as well as time spent in the tailored modules. There was no interaction between stage of change and time spent in the tailored modules.

## Discussion

### Principle Results and Comparison With Prior Work

In the present study, there were significant increases in self-reported physical activity levels between baseline and the last follow-up after 13 months in all participants, but there were no significant differences between the randomized groups. More pronounced increases were found in SU of Active-online. However, these individuals were not randomized and thus cannot be directly compared with the randomized groups. Furthermore, SU willing to participate in the study may not be representative of all Active-online users since they were a self-selected sample and only represented 7.4% of all visits on Active-online during the recruitment period.

Self-reported changes in physical activity levels were not confirmed by objective measures. Differences between self-reported and objective measures may be due to the possibility that study participation influenced the perception of physical activity behavior and thus reporting of physical activity levels. A seasonal pattern [43], with lower activity levels in winter (FU2), was observed in both self-reported and objective physical activity data.

Results of other computer-tailored [10] and Web-based tailored [23,24] physical activity intervention studies have been mixed. The results in the present study are comparable with other studies investigating Web-based physical activity interventions. While some studies produced effective results in the short term [20,21] or when compared to a waiting list control group [26], others showed improvements in physical activity levels in both intervention and control conditions [17], like we did with regard to self-reported physical activity. A tailored intervention that has been effective when delivered on CD-ROM in a controlled setting after 6 months [44] and after 2 years [22] was not effective when delivered online in a real-life setting compared with online standard advice [25]. Similar to our study, Spittaels et al also found increases in self-reported physical activity levels in both intervention and control groups, and increases in physical activity levels were not confirmed by accelerometer data [25]. The present study adds evidence to the point that effectiveness of a Web-based physical activity intervention may be difficult to demonstrate when delivered in an uncontrolled setting.

As per the real-life setting, study participants were free to start and stop the intervention. In addition, the anonymous nature of the Internet and the wealth of available information may make it difficult to achieve sufficient levels of intervention use. On average, individuals in the IG and SU started a tailored module less than twice during the study period, accumulating a mean of 23 minutes in the tailored modules in total (12 minutes per visit). In a study assessing user attitudes toward a physical activity website, an average time of 7.1 minutes spent on the tailored intervention per visit and a total average of 356 minutes over 1 year was reported [45]. While the duration per visit was higher in our study sample, the total accumulated time spent on the intervention was 15 times higher in the other study. Leslie et al reported that participants who entered a tailored Web-based physical activity intervention spent, on average, 9 minutes per visit [46]. However, 152 participants produced 4114 visits on the website over 8 weeks, indicating that the accumulated exposure was clearly higher in that study than in our sample. Low exposure to intervention materials has been reported in other studies using objective data on website usage, indicating that achieving engagement in website-delivered physical activity interventions is challenging [23]. Moreover, one quarter of the participants in the IG and SU did not start a tailored module at all, and 13.7% of controls used Active-online independently of the study, suggesting some degree of contamination in the IG and CG. This may have reduced a potential effect but reflects the real-life delivery mode used in this study. While correlations between log-in frequency and weight change have been reported in a study focusing on a Web-based behavioral weight loss program [47], the role of frequency and duration of use of Active-online on changes in physical activity behavior could not be clarified in this study.

Because of the challenges that we face with stand-alone Web-based interventions that are freely accessible on the Internet, it may be more promising to embed a program like Active-online in a wider context of health promotion. Possibilities for better utilization of Active-online may be its application in a workplace setting, the “prescription” of Active-online to patients in primary care, or the inclusion of Active-online in a larger health promotion packet targeting different health issues, for example, in a community setting. Two studies that have evaluated Web-based tailored interventions in a primary care setting have reported increases in physical activity levels after 1 month [48] and after 6 weeks [49]. A study in two manufacturing and two office sites showed high levels of engagement in a Web-based and monitoring device–based physical activity and weight management program in a wide range of employees [50]. A computer-tailored (but not Web-based) intervention for nutrition and physical activity in a workplace setting demonstrated increases in the frequency of strengthening and flexibility exercise compared to a delayed group [11]. A Web-based workplace health promotion program targeting nutrition, stress, and physical activity did not outperform print materials used in the control group, even though improvements in some physical activity variables were reported in both groups [51]. Further research may investigate possibilities of integrating Web-based interventions in a wider health promotion context. Marcus et al especially highlight the urgent need for research on Internet-based physical activity programs within the context of primary care [52].

### Strengths and Limitations

A strength of the study was the delivery of the Web-based intervention under real-life conditions, not in a controlled setting. There were no face-to-face contacts or other factors that may increase compliance, because they do not represent realistic conditions for open-access Web-based interventions. Furthermore, objective physical activity assessment was used in a subsample of participants in addition to the questionnaires. We included SU of Active-online as an additional study arm. Frequency and duration of use of Active-online were monitored using objective data from the Active-online user database, making it possible to look at the relation between use of Active-online and physical activity changes. Other strengths are the long-term follow-up and the large number of participants included in the randomized study.

Several reasons may be responsible for the limited effectiveness of Active-online. The website was tested in 2003 and acceptability was generally good; participants especially liked the individual counseling, the pleasant tone, and the simple structure and design [29]. However, Internet technology is changing rapidly and Active-online may already be slightly out-of-date. Furthermore, Active-online is based on the transtheoretical model of behavior change, which was regarded as promising at the time when Active-online was developed, but has more recently been subject to some debate regarding its potential to change behavior [53,54]. In addition, baseline physical activity levels were already quite high in the study sample, with around 280 minutes total reported activity time per week; thus a ceiling effect may have occurred.

This study has several limitations. A rather low overall response of around 50%, as observed in other studies [30], was expected based on the experiences of the feasibility study [32] and was taken into account when calculating the sample size. High drop-out attrition has been recognized as a common problem in Internet-based studies [55]. Nonresponse in this study was differential between groups, with higher drop-outs in the IG and SU than in the CG. Differences between responders and nonresponders [30] and higher drop-out rates in intervention groups have been observed in other studies in the domain of physical activity [20] and nutrition [56,57]. The smaller number of recruited SU and the fact that they only represented 7.4% of all visits on Active-online during the recruitment period limits conclusions about the effectiveness of Active-online in this group. The time spent on Active-online recorded in the database may not represent the actual time spent interacting with the intervention, because Active-online may have been opened in the background while the user was browsing other websites opened simultaneously. On the other hand, Active-online users could print their feedback reports and read them offline. If study participants revisited Active-online without using their password, the estimated number of visits presented here may be conservative. Last, due to a technical problem, the reminder emails to revisit Active-online were not sent out according to the original schedule for registered users of Active-online at 2, 4, and 7 months.

### Conclusions

The present study showed limited effectiveness of Active-online in a randomized sample of volunteers from the general adult population when offered as a stand-alone intervention delivered online under real-life conditions. Further research may investigate the potential of Web-based physical activity interventions integrated in a wider context, for example, primary care or workplace health promotion.

## Multimedia Appendix

Application for funding

## Abbreviations

BMI: body mass index
CG: control group
CI: confidence interval
FU: follow-up
HEPA: health-enhancing physical activity
IG: intervention group
OR: odds ratio
SU: spontaneous users
