# Supplementary material for: Effectiveness of Active-Online, an Individually Tailored Physical Activity Intervention, in a Real-Life Setting: Randomized Controlled Trial
Source: J Med Internet Res. 2009 Jul 28;11(3):e23. doi: 10.2196/jmir.1179 (PMC2763402; doi:10.2196/jmir.1179)
Supplement: Supplementary file 1 [file jmir_v11i3e23_app1.pdf]

Eidgenössische Sportkommission *ESK*
Commission fédérale de sport *CFS*
Commissione federale dello sport *CFS*
Cumissiun federala da sport *CFS*
Federal Council of Sports *FCS*

BASPO *Bundesamt für Sport Magglingen*
OFSPO *Office fédéral du sport Macolin*
UFSPO *Ufficio federale dello sport Macolin*
UFSPO *Uffizi federal da sport Magglingen*

FORSCHUNGSGESUCH

Zum Formulieren eines Forschungsgesuches benützen Sie bitte die „Erläuterungen für das Ausfüllen des Formulars Forschungsgesuch".[*)]

Die Gesuche sind einzureichen beim:

BUNDESAMT FÜR SPORT
Sportwissenschaftliches Institut
Forschungskonzept "Sport und Bewegung“ 2004-2007
CH - 2532 Magglingen

[*)] Rechtsgrundlage: Wegleitung vom 22. Januar 1999 für Beiträge an sportwissenschaftliche Forschungsprojekte

Sekretariat/Secrétariat/Segretariato, CH - 2532 Magglingen/Macolin
Tel.: ++41(0)32/327 63 27
Fax: ++41(0)32/327 64 05
e-mail: esther.puma@baspo.admin.ch

ZUSAMMENFASSUNG DES FORSCHUNGSGESUCHES
(max. 1 Seite, d.h. 3'000 Zeichen)

(Hintergrund und Kontext, Fragestellung, Methode, erwartete Resultate, Relevanz)

Hauptgesuchsteller/Hauptgesuchstellerin:
Dr. med. Brian Martin, MPH
Kurztitel:
Effectiveness of active-online.ch: A randomised controlled study

---

Zusammenfassung:

Introduction: www.active-online.ch is a freely available interactive website, offering an individually tailored counselling and motivation program for individuals between about 30 and 60 years of age who are not active on a regular basis. It is based on the Transtheoretical Model. An evaluation revealed that the target population could be reached and that the structure and design of the website were well accepted. The program is currently disseminated nation wide in Switzerland. The study aims to assess how effective active-online.ch is before it is more heavily promoted or possibly adapted to specific target groups.
In 2003, the feasibility of an internet-based randomised controlled trial to study the effectiveness of the program to change physical activity behaviour was tested in a pilot study. The study was co-financed by the Federal Council of Sports FCS with 70’000.- CHF. It demonstrated that such a design is feasible: The target population of active-online.ch could be reached with multimedia recruitment; loss during questionnaire administration and loss to follow up could be quantified in dimensions that can be accommodated by study design; realistic estimates of the number of participants which could be recruited with advertisements and through Email announcements in an enterprise were yielded. Most recent developments in internet technology have to be taken into account.
Study questions: The main study question is whether the individually tailored intervention program active-online.ch is more effective to change knowledge, attitudes and behaviour regarding physical activity than a standard intervention (internet-version of a flyer recommending more physical activity).

Methods: The effectiveness of the program will be assessed with an internet-based randomised controlled trial. Subjects will be recruited trough calls in the media. In the preparation period of the study we will investigate whether it will be possible to recruit enough companies or administrative units to additionally conduct a quasi-experimental study arm in a workplace setting. In this case, the companies will be randomised or matched to the intervention or control conditions. Subjects within the companies will be recruited by Email.

Expected results: It is expected that larger positive changes in knowledge, attitudes and physical activity behaviour can be observed among individuals that were randomised to the individualised program active-online.ch than among individuals randomised to the standard intervention.

Relevance of the study: First experiences with the dissemination of active-online.ch demonstrate that the program is well accepted and used by a relevant proportion of the Swiss population. The potential for active-online.ch as an intervention to promote physical activity in Switzerland is therefore considerable and the investigation of its effectiveness is essential to estimate its Public Health impact to plan and justify the investment of resources into this or similar programs in Switzerland. On the national as well as on the international level there are no studies so far assessing the effectiveness of internet-based tailored intervention programs for physical activity or any other health behaviour. Therefore, a study on the effectiveness of active-online.ch will also provide results relevant for the international research and public health community. A study on the effectiveness of active-online.ch to change physical activity behaviour fits well into the first priority (*Promotion of health through physical activity and sports)* of the research sector *Sport and Physical Activity* of the *Research in Swiss Government Departments* 2004 – 2007.

Allgemeine Angaben

1.1 Titel des Projekts:

Internet-based randomised controlled study on the effectiveness of the physical activity program active-online.ch

1.2 Kurztitel: (max. 50 Zeichen)

Effectiveness of active-online.ch: A randomised controlled study

1.3 Wissenschaftliche Fachgebiete:

Evaluation, Health Promotion, Prevention, Epidemiology

1.4 Hauptgesuchsteller/Hauptgesuchstellerin:

Name: **Martin** Vorname: **Brian**
Akadem. Grad: **Dr. med., MPH** Geburtsdatum: **21. 8. 1962**
Stellung: **Head of the Health Promotion Unit**
AHV-Nr.: **633.62.352.411** Nationalität: **Swiss**

Institution: **Institute of Sports Sciences, Federal Office of Sports**

Adresse Institution: **Bundesamt für Sport, Sportwissenschaftliches Institut, 2532 Magglingen**

Telefon: **032 327 62 38** Fax: **032 327 64 05**
e-mail: **brian.martin@baspo.admin.ch**

1.5 Beantragter Betrag: Fr. **186'980.-**

1.6 Konto-Nummer für die Überweisung (Postscheck oder Bankkonto):
Postcheck-Nr.: Bank:
Ort:
Konto-Nr.:

1.7 Dauer (Beginn, Abschluss): **January 2005 – December 2006**

1.8 Mitgesuchsteller/Mitgesuchstellerin (fakultativ):

Name: **Bauer** Vorname: **Georg**
Akadem. Grad: **Dr. med., DrPH** Geburtsdatum: **9. 2. 1962**
Stellung: **Head of the Department for Health and Intervention Research**
AHV-Nr.: **143.62.140.154** Nationalität: **German**

Institution: **Institute for Social and Preventive Medicine, University of Zurich**

Adresse Institution: **Institut für Sozial- und Präventivmedizin der Universität Zürich, Abteilung Gesundheits- und Interventionsforschung, Sumatrastrasse 30, CH-8006 Zürich**

Telefon: **01 634 46 38** Fax: **01 634 49 62**
e-mail: **gfbauer@ifspm.unizh.ch**

2. Wissenschaftliche Angaben

2.1 Forschungszielsetzung

2.1.1. Rationale of the study

a) physical activity and health

The outstanding importance of physical activity as a health resource and for the prevention of various chronic diseases has been documented extensively (Marti & Hättich, 1999; U.S. Department of Health and Human Services, 1996). In the last decade, the recommendations for health enhancing physical activity (HEPA) have focussed on moderate intensity physical activity as part of an active lifestyle. In Switzerland, national authorities have adapted the international recommendations for health enhancing physical activity, which are defined as the accumulation of 30 minutes or more of moderate intensity activities on most, preferably all days of the week, or alternatively at least 20 minutes of vigorous exercise on three or more days of the week (Bundesamt für Sport (BASPO) et al., 2002). Nevertheless, at least one third of the Swiss adult population does not meet any one of these two recommendations (Martin et al., 1999; Martin 2002), which causes 1.4 million cases of disease annually, almost 2'000 deaths and direct treatment costs of 1.6 billion Swiss francs (Martin et al., 2001). In Switzerland, the need for interventions to promote physical activity has been recognized and has influenced the political agenda-setting (The Swiss Federal Government's Concept for a National Sports Policy, November 30th 2000).

b) Tailored interventions using the TTM

The Transtheoretical Model of behaviour change (TTM, Prochaska et al., 1992) is a theoretical framework which describes how individuals change their behaviour. The model postulates five distinct stages which are passed during individual behaviour change: in the precontemplation stage, a person has no intention to change a problem behaviour, in the contemplation stage, a person intends to change his or her behaviour within the next six month, in the preparation stage, a person tries the new behaviour and intends to change it within the next month, in the action stage, a person has adopted a new behaviour less than six months ago, and in the maintenance stage, a person has successfully changed a problem behaviour more than 6 months ago. In any stage relapse to a previous stage is possible, but any relapse increases the possibility to reach the maintenance stage in the next attempt. Prochaska et al. (1992) found ten strategies (called "processes of change") which individuals selectively use while passing the five stages of change. The use of cognitive strategies like consciousness raising or self-reevaluation in early stages and the use of behavioural strategies like stimulus control or reinforcement management in later stages increase the probability of successfully changing one's behaviour (Prochaska et al., 1992). During behaviour change, self-efficacy and the perceived advantages ("pros") of behaviour change increase, while the perceived disadvantages ("cons") decrease.

In the last decade, the Transtheoretical Model has often been used as the theoretical framework for individually tailored interventions for behaviour change (overview Kreuter et al., 2000). These interventions are computer based and counselling information is tailored to the individual characteristics of the participants, simulating a one-to-one counselling situation where counsellors ask questions and use the answers to determine the most appropriate course of action for that particular individual, given his or her unique life circumstances. Using computer technology, individual counselling can be provided for a large number of people.

A variety of such tailored intervention programs has already been developed, particularly in smoking cessation (Dijkstra et al., 1998; Dijkstra et al., 1999; Etter & Perneger, 2001; Martin-Diener et al., 1997; Martin-Diener et al., 1999; Prochaska et al., 1993; Strecher, 1999; Velicer et al., 1993), but also in nutrition (Brug et al., 1996; Brug et al., 1999; Campbell et al., 1994, Campbell et al., 1999; Oenema et al., 2001), and in the

promotion of physical activity or exercise (Bull et al., 1999; Marcus, Bock et al., 1998; Sciamanna et al., 2002; Napolitano et al., 2003; Vandelanotte & De Bourdeauhuij 2003; Sciamanna, Marcus et al., 2004, Sciamanna, Novak et al., 2004). Particularly in smoking cessation, these programs have proven to be effective (Lancaster & Stead, 2002). A detailed review on the effectiveness of physical activity programs will be given in section 2.1.2 (short state of the art). In most published studies, the tailored intervention material was created electronically, but the questionnaires as well as the feedback material were distributed using the postal way. In only two projects so far, the Internet was used to collect information and distribute individualized feedback (Oenema et al, 2001; Sciamanna et al., 2002 and Napolitano et al., 2003), as this is the case in the program active-online.ch which will be introduced below. In another study, stage-matched material was collected and distributed via Internet (Marshall, Leslie et al., 2003). Furthermore, the feasibility of incorporating computer-tailored physical activity counselling in primary care settings (Sciamanna, Marcus et al. 2004) and patient satisfaction with this new services (Sciamanna, Novak et al., 2004) is under investigation.

There is growing interest in such tailored intervention programs in Public Health for two reasons: Because many of these programs have shown to be more effective than standard intervention programs, and because the use of computer technology and the Internet makes it possible to reach a large number of individuals at low costs. Data provided by the Federal Office of Statistics (Bundesamt für Statistik et al., 2002) revealed that in 2001, 50% of the Swiss population used the Internet at least once in the past six months, and over 33% used it several times per week. Considering the upward trend of Internet use over the previous five years, it must be assumed that these figures will continue to increase.

c) Description of the counselling program active-online.ch

www.active-online.ch is a freely available interactive website, offering an individually tailored counselling and motivation program for individuals between about 30 and 60 years of age who are not active on a regular basis. It is based on the Transtheoretical Model. An evaluation in late 1999 revealed that the target population could be reached and that the structure and design of the website were well accepted (Martin-Diener & Thüring, 2001; Martin-Diener, Thüring & Bauer, manuscript submitted).

In the main section of the program active-online.ch, participants answer one to four online-questionnaires, so their current stage of change, the pros and cons for change, self-efficacy, and the use of processes of change can be assessed. After each questionnaire, a motivational feedback tailored to the individual characteristics is displayed immediately. Completely inactive participants are motivated to start with any moderate intensity physical activity. Depending on their preferences, occasionally active persons are motivated either for regular moderate intensity or for regular vigorous intensity activities. Participants can compare themselves with other participants in the same stage and with their own situation during their last visit in the program. The latter requires online ordering of a personal password. Finally, participants can subscribe for bimonthly supporting e-mails.

The program was developed between 1999 and 2002 at the Institute for Social and Preventive Medicine, University of Zurich, and at the Institute for Sport Sciences, Federal Office of Sports, Magglingen, by an multidisciplinary team of experts in Public Health, Sport Sciences, Psychology, Design and Computer Sciences. It was developed in German and then translated into French and Italian. The project was funded (total costs: CHF 800’000.-) by five national partners (Health Promotion Switzerland, the Federal Office of Sports, the Swiss Accident Insurance Company suva, Allez-hop and Qualitop).

d) Dissemination of active-online.ch

The program was officially launched in April 2003 with a media event. It is currently disseminated mainly in collaboration with the largest Swiss internet portal (bluewin). Furthermore, the project partners use their specific channels to distribute the program. The hosting and monitoring of the program is financed by the partners who developed it, with the Federal Office of Sports being mainly responsible.
From April 2003 until July 2004 about 155'000 visits were counted on the website, 70% originating from users in Switzerland (Swiss population: 7 millions). About 40% of all visitors entered via the bluwin-hompage. In the same time period, some 40'000 participants actually started the interactive program and 28'000 progressed at least to the feedback regarding their current stage of change. 60% of the participants starting the interactive program finished it.

e) Need for the study of effectiveness

In order to optimise the dissemination of interventions promoting physical activity through the Internet, program evaluation is essential. Effectiveness evaluation of a program is not only fundamental for the quantification of the Public Health impact of the intervention, but especially for the evidence-based planning, development and implementation of new or adapted intervention programs. The results of an effectiveness evaluation of active-online.ch will strongly influence the amount of resources that will be invested into the dissemination of active-online.ch on the population level.
A study of effectiveness will ideally evaluate a program in its naturalistic setting: Therefore, communication channels and subject recruitment for the effectiveness study should be comparable to the dissemination strategies planned for that particular program. On the international level, no internet-based tailored intervention program has been evaluated for its effectiveness so far (see section state of the art below). This study will therefore be of great importance also for the international research community interested in the field.
The Internet not only opens new channels for the distribution of health information, but as a platform for subject recruitment and data collection also allows for new study designs and methods in evaluation research. The knowledge and evidence in this area of evaluation research is still scarce, although Internet-based research becomes more prominent in various fields (Batinic et al., 1999).

2.1.2. Short state of the art of effectiveness evaluation of tailored interventions to promote physical activity

Randomized trials (RCT) investigating the effectiveness of tailored interventions to increase physical activity have been undertaken mainly by Bess Marcus and collaborators (Marcus, Bock et al., 1998; Marcus, Emmons et al., 1998). The main interest of these studies was to demonstrate superiority of tailored print material over standard material, and outcome measures were stage progression in the TTM model. Marshall, Bauman et al. (2003) and Marshall et al. (2004) investigated the effectiveness of stage matched intervention material in population –based RCTs and Marshall, Leslie et al (2003) compared the effectiveness of print- and internet-versions of a stage-matched program in an RCT. Other interventional studies investigated the effectiveness of physical activity counselling in primary care (Eakin et al., 2000), of programs for special clinical or non-clinical target groups or of exercise programs in schools, worksites and other settings (overview Sallis & Owen, 1999). Although positive effects could be found in most of the studies, the reported effect sizes strongly depended on the intervention and the outcome measures used. The studies are often non-randomised and therefore prone to bias.
There is one first preliminary RCT evaluating user attitudes (Sciamanna et al., 2002) and the behavioural effects (Napolitano et al., 2003) of an internet-based physical activity intervention: Subjects in the intervention group (N=30) were more likely to have progressed in the stages of change than individuals in the control group (no

intervention, N=35) after one and three month. The intervention group was active with moderate intensity longer than the control group after one, but not after three month.

2.1.3. Results and conclusions of the feasibility study for an effectiveness evaluation of active-online.ch

A feasibility study to conduct an internet-based randomised controlled trial to investigate the effectiveness of active-online.ch in a real life setting was conducted from April 2003 until March 2004. The study was co-financed by the Federal Council of Sports FCS with 70'000.- CHF.

Self-selected participants were recruited for a "health study" using various media channels (print advertisements, Internet banners, e-mail in a federal administration unit). From the 321 individuals who had answered an online questionnaire, 132 (41%) had to be excluded from the trial because of indications of a potential health risk in the physical activity readiness questionnaire PAR-Q. Another 18 of the potential participants (6%) stopped when their e-mail address was demanded. 171 subjects were thus randomised into an intervention (n=97) and a control group (n=74) and re-contacted by e-mail after six weeks and six months to answer an online follow-up questionnaire. E-mail recruitment and one of the print advertisements were most effective to recruit study participants. Response after six weeks was 76%, complete data of all three measurements over six month are available from 63% of the participants. The participants who quit the online questionnaire prematurely were significantly more often women, married and reported better subjective health than individuals willing to participate. Non-respondents after six months were significantly more often women, smokers and less educated than respondents. 57.4% of the recruited participants were physically inactive. This corresponds to the proportion of inactive persons in the Swiss population. More detailed information is given in the complete report of the feasibility study (Thüring, Martin-Diener, Martin & Bauer, manuscript submitted).

The main conclusions of this study are:

- **Internet technology is convenient and useful for conducting a randomized effectiveness study – if adapted to the latest developments.**

  No major technical problems in programming the study website, with the randomization or in the e-mail delivery system were encountered. About 20% of the participants in the intervention group did not receive the intervention because they used Internet security software which prevents automatic forwarding to other Internet addresses as used in this study. This problem can be resolved by hosting the questionnaire and the intervention program under the same address. With the latest software developments, automatic e-mailing as was used in this study could become a problem because spam filters identify the study messages as junk e-mail and do not deliver them.

  The pilot study revealed as well, that the possibility to react quickly and adapt to obvious shortcomings in data collection is one major advantage of an Internet or computer based study. It was very reasonable to test the Internet applications in a pilot study to eliminate potential threats to data validity in a full scale study of effectiveness.

- **There are effective multimedia strategies to recruit participants required for a full scale study of effectiveness.**

  The most effective and cost-effective method to recruit participants was the e-mailing to employees of a Federal administration unit. The fact that a member of the top management sent the e-mail and invited the employees to participate has surely contributed to the high participation of about almost 50%. Despite its very small size, the print advertisement in a tabloid Sunday paper was an effective way to recruit participants. Its costs of about 14.- Euro per individual willing to participate reach sensible dimensions.

The Internet banners were neither cost-effective (26.- and 35.- Euro respectively per individual willing to participate) nor a successful way of recruiting a large number of participants, although the clicking rates on our banners were not worse than for comparable commercial ones [personal communications from the marketing departments of the two websites].

- **The target population of active-online.ch can be reached with an online-study.**

  A majority of the participants belongs to the target group of active-online.ch defined as not sufficiently active persons between 30 and 60 years of age. The proactive recruitment strategy by e-mail reached men and women equally, more women participate via the other channels. Persons with a university degree and a higher vocational training are clearly over-represented in this study, as among Internet users in general (Huber, Cosandey, & Täube, 2002) as well as among the employees of the involved unit of the federal administration. The participants in this study did not have extreme health characteristics that would bias the results of an effectiveness study.

- **Loss during questionnaire administration and loss to follow-up is not a major problem**

  Only 6% of the participants who answered the online questionnaire refused to register their e-mail address. No fictional addresses like “aa@bb.cc” were detected among the 3.5% undeliverable e-mails after six weeks, the main reason for the delivery failure seemed to be spelling errors. More than three quarters of the participants in the trial responded to the e-mail call after six weeks, despite the open setting of the Internet. Participation after six months is over 83%. Overall, 63% of all subjects participated in both follow-up measurements. Once registered in the study, the majority of the self-selected participants is willing to invest some more time in answering the questions a second and a third time. The fact that 5.3% of all participants in the trial responded after six months but not after six weeks shows that for a future study of effectiveness, it should be considered to re-contact those participants who have not responded to the e-mails in order to increase the response.

- **The PAR-Q is not suitable for risk screening in an Internet application**

  If applied without further context information or instructions, the PAR-Q (Physical activity risk questionnaire, ACSM 1998) does not seem to be suitable for recruitment of participants in the context of an online study. Its high sensitivity and the low specificity can create problems when used for excluding participants. In a future study of effectiveness of active-online.ch, other ways of excluding participants with major health risks or problems must be considered. However, in the intervention program active-online.ch, all participants are reminded to screen for potential health risks when starting with physical activity and the PAR-Q is also provided.

- **As estimated, about 1000 study participants will be needed to detect significant differences in physical activity behaviour.**

  As expected, no significant effect on physical activity behaviour could be detected in the feasibility study due to the small sample size. Nevertheless we found that more persons in the intervention group became active after six weeks than in the control group (14% versus 10%) and in the intervention group less individuals relapsed than in the control group (6% versus 9%). To detect significant group differences with distributions we found in this study, at least 1000 participants will be needed. See section 2.2.2 for details regarding sample size calculations.

2.1.4. Research questions

The study adresses three main research questions. The first questions relates to the effectiveness of active-online. Effectiveness among independent users recruited by mass media are compared to the effectiveness among groups of users recruited by their common worksite. This is an important issue because promotion of active-online through worksites could be efficient if the program proves to be effective in this context. The second research questions aims to assess if the results produced by participants of a particular study website can be generalized to the spontaneous users of active-online. Based on this information, the third questions adresses the overall public health impact of active-online on the target population.

1a) How effective is active-online.ch to change physical activity behaviour, knowledge about and attitudes towards health enhancing physical activity?

- Is the individually tailored physical activity promotion program more effective to increase knowledge about health enhancing physical activity than a standardised intervention?
- Is the program more effective to increase positive attitudes towards physical activity than the standard intervention?
- Is the program active-online.ch more effective to change physical activity behaviour than a standardised physical activity intervention?

1b) To what extent can the effectiveness of active-online among independent users recruited by mass media be compared to the effectiveness among groups of users recruited by their common worksite?

- At baseline, do users of the study website recruited by mass media differ from users recruited by their worksite regarding age, gender and physical activity behavior?
- At follow-up, do users of the study website recruited by their worksite become more active than users recruited by mass media?

2) To what extent can the effectiveness of the program to change behaviour assessed in an outcome study (research question 1) be compared to the effectiveness of the program among spontaneous users?

- At baseline, do participants in the controlled study differ from spontaneous visitors to active-online.ch regarding age, gender and physical activity behaviour?
- Do spontaneous visitors of the website active-online.ch become more active after participating in the program compared to users of the controlled study?

3) What is the public health impact of active-online.ch?

- How many individuals currently use spontaneously the regular active-online.ch webpage in the context of the current implementation process?
- What is the overall estimated impact of active-online.ch on the physical activity behaviour of these users considering the results of the effectiveness study?

2.2 Forschungsmethode

2.2.1. Study design and research plan

a) Research question 1: Effectiveness of the program

To study the effectiveness of active-online.ch to change behaviour, knowledge or attitudes, two study arms with two different designs are planned: 1) a randomised controlled trial (RCT) with individuals recruited trough the media, and – if possible - 2) a quasi-

experimental study with companies or other organisational units. During the first two month of the preparation period it has to be estimated whether it will be possible to recruit the number of employees needed (sample size considerations see below). If these investigations are promising, the quasi-experimental study arm will be realized; if not, this arm will have to be dropped.

- Randomised controlled trial
Participants will be randomised on the individual level and assigned either to active-online.ch or to a web-based standardised physical activity program (*figure 1*). The standardised control intervention corresponds to a web-version of a flyer recommending more physical activity. It will also provide the opportunity to do the PARQ, as it is the case in the program active-online.ch. The procedure of the study will be analogous to the pilot study described above.

- Quasi-experimental study
Members of companies or other organisational units will be (randomly) assigned on the group level to either active-online.ch or the standard intervention (*figure2*). The study procedure is comparable to the pilot study and the RCT arm described above.

In both studies, participants will fill in the baseline assessment online and will then be forewarded to either active-online.ch or the control intervention. The control group will receive a short standard intervention, recommending participants to become more physically active.

If both study arms are possible, effects will be measured separately. If the effects in both study arms are comparable and if the members of the two study groups do not differ, data may be pooled to analyse effects in subgroups (e.g. gender, stages of change).

b) Research question 2: Comparison with spontaneous users

- follow-up of spontaneous visitors to the website
During two month, spontaneous visitors to active-online.ch will be asked on the homepage to participate in a study on active-online.ch. If they agree, they will receive the same procedure as the intervention groups of the controlled study arms. Users who do not want to participate in the study will be able to visit without any further questions. There will be no control group. The comparison of the outcomes of interest between the actively recruited intervention groups and the spontaneous visitors will allow to generalize the results of the controlled study to the spontaneous use of the website.

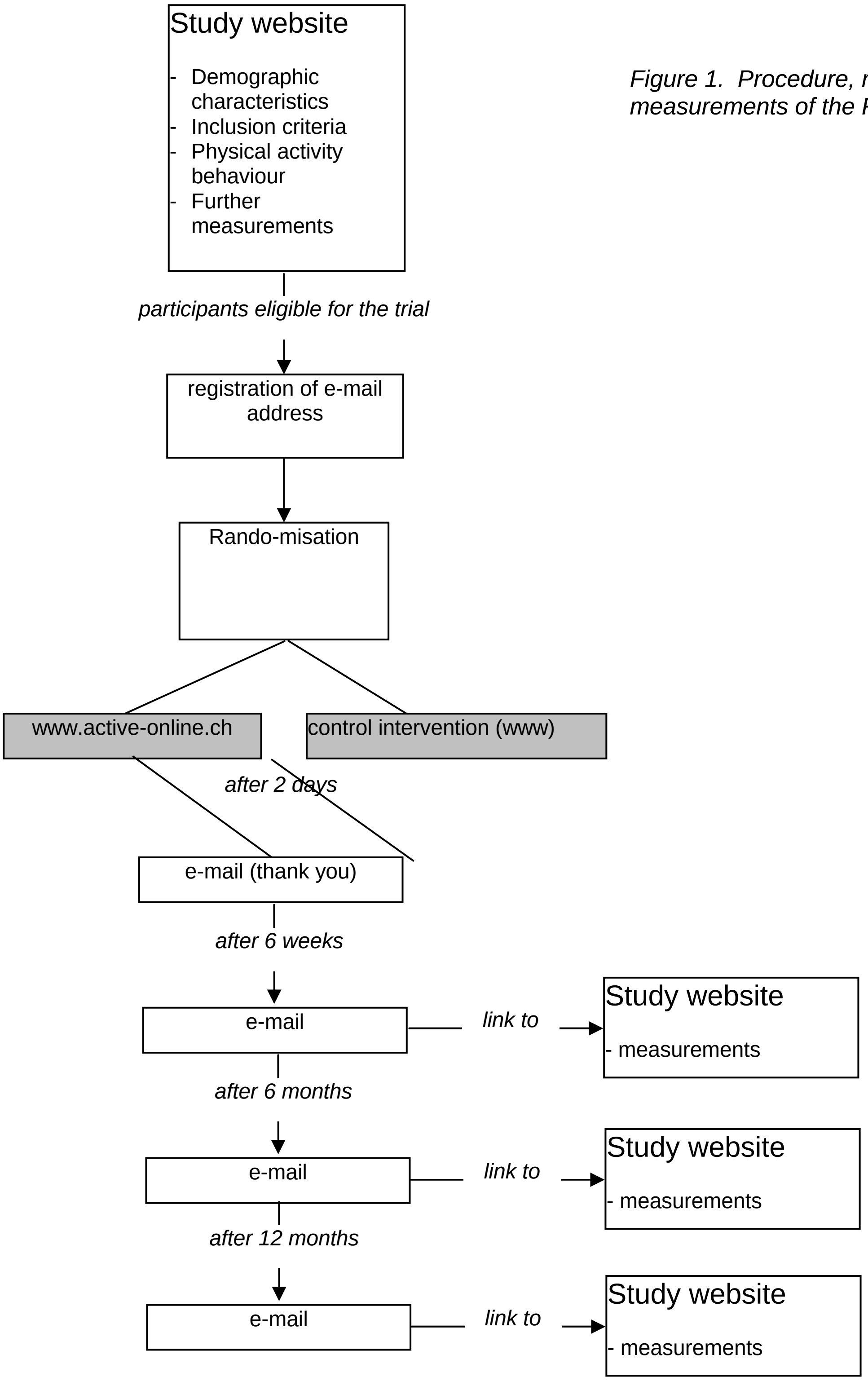

Figure 1. Procedure, randomisation, follow-up measurements of the RCT.

Figure 2. Procedure, randomisation, follow-up measurements of the quasi-experimental study.

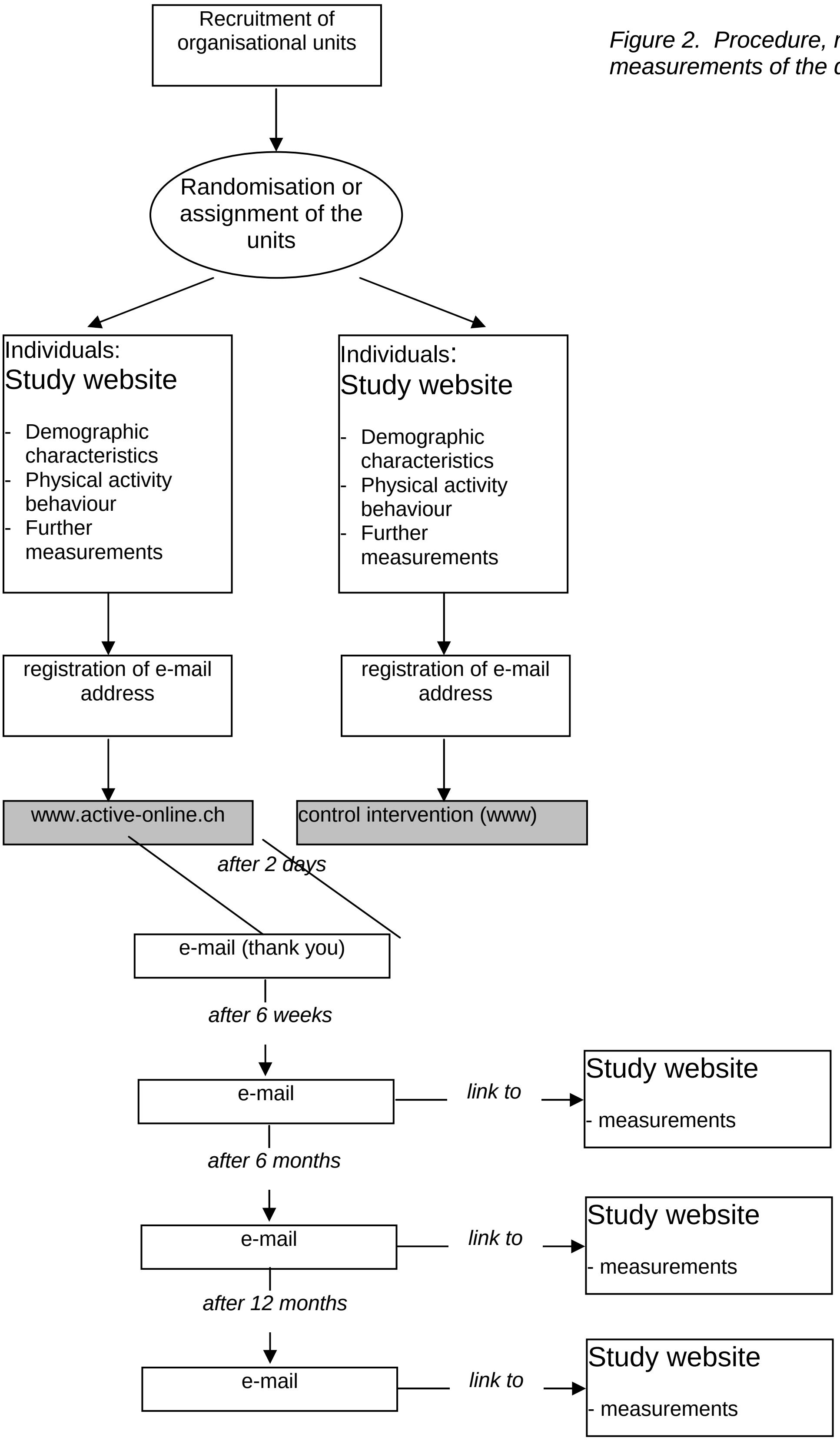

c) Research Question 3: Public Health Impact

- analysis of the data base of active-online.ch

From the data of spontaneous users that is recorded in the expert system the number of individuals that have used active-online.ch in a given time span and their characteristics will be determined. The Public Health Impact will be estimated by multiplying the effectiveness of the program (results of research question 1) with the proportion of the population that is reached in the context of the implementation process.

2.2.2. Study population, sampling and sample size

a) Research question 1: Effectiveness of the program

- randomised controlled trial

The *study base* is the German speaking population of Switzerland who uses the internet. Participants will be recruited through announcements in print media (e.g. Sonntagsblick) for a study on physical activity and health conducted by the Institute for Social and Preventive Medicine of the University of Zurich. People will be invited to visit the website www.online-studie.ch to participate. The pilot study has demonstrated that the Physical Activity Readiness Questionnaire’s (PARQ) high sensitivity and low specificity make it impracticable tool for excluding participants with a potential health risk from the study. Therefore, no participants will be excluded from the study basing on a risk screening procedure. However, the PARQ is an element of the program active-online.ch itself, recommending participants with a potential health risk to look for professional advice. The control intervention will also offer the same procedure. Thus, all individuals completing the baseline questionnaire and willing to give their Email-address will be included in the study.

Sample size: Studies of interventions to increase physical activity differ in the reported effect sizes, which are strongly dependent on the outcome measures used in the studies (Dishman & Buckworth, 1996). According to a meta-analysis of the Transtheoretical Model applied to physical activity (Marshall & Biddle, 2001), an individualized and stage-specific physical activity counselling intervention like active-online.ch can be expected to have an intermediate to strong behavioural efficacy. Because in the program active-online.ch the counselling takes place on the Internet, i.e. in a natural and uncontrolled setting, its effect must be expected to be smaller than in more controlled intervention settings. To detect a small behavioural effect of active-online.ch (assumption: 30% of the individuals in the intervention group become regularly active and 20% of the individuals in the control group) with 80% probability, complete data on about 800 to 1000 subjects including the intervention and control group must be available. Taking into account loss to follow up of 30-40% (observed in the pilot study), a total of 1500 (-2000) individuals must be randomised to either the control or the intervention group at baseline.

- quasi-experimental study

The *study base* are all members of the recruited organisational units who routinely use the internet (i.e. have an Email address in their organisation). It is planned to ask for participation either several units of the federal administration or different branches of larger companies such as banks or insurances. Within the organisational units, individuals will be recruited by Email. The inclusion criteria correspond to the RCT arm.

Sample size: As in the RCT, about 1500 subjects have to participate in the study. Assuming a participation of 40% of the contacted individuals (pilot study 50%), organ-

isational units with a total of at least 4000 employees with access to the internet will have to be recruited.

b) Research question 2: Comparison with spontaneous users

- follow-up of spontaneous visitors of active-online.ch
The *study base* are all the visitors of the German version of active-online.ch. The inclusion criteria correspond to the RCT arm.

Sample size: Participants will be recruited during two months. We have no experience so far, regarding the proportion of visitors that will agree to participate in a study. Since about 10'000 people visit the website per month it should be possible to recruit a number of participants comparable to the controlled study arms within two month. The period will be extended to four months if within two months only an insufficient number of participants can be recruited.

c) Research question 3: Public Health Impact

- analysis of the data base of active-online.ch
To determine the proportion of the population that has used active-online.ch, all participants who have visited the program spontaneously will be included in the analyses.

2.2.3. Measurements

a) Research questions 1 and 2: Effectiveness and comparison with spontaneous users

Measurements at baseline only

- Access to study website (channel used)
- Demographic characteristics: age, gender, family status, educational level, adapted from the Swiss Health Survey
- height

Measurements at baseline and at follow-up

- caloric expenditure: short version of the physical activity frequency questionnaire (Bernstein et al., 1998).
- physical activity behaviour and stage of change (Mäder et al., 2002; Martin, 2002)
- knowledge about physical activity recommendations from the Swiss hepa-Survey (Martin, 2002)
- Self-efficacy (Marcus et al., 1992)
- smoking habits
- weight
- Health related quality of life: SF12 (Bulliger & Kirchberger, 1998)

Accelerometry
If the quasi-experimental study arm can be realised, a sub-sample of the study participants will receive an accelerometer before baseline assessment and at follow up (about 50 subjects of the intervention and 50 of the control group). Caloric expenditure will be assessed.

b) Research question 3: Public Health impact

To estimate the proportion of the population reached by active-online.ch the data base of the program will be analysed. The criterion for a “reached individual” must be comparable to the intensity at which the program was actually used in the outcome studies (research question 1): For example, if half of the participants in the study finish the program, and the other half exits prematurely or does not even start the program, a comparable behaviour towards the program must be allowed also when counting the individuals that “were reached” in the real life dissemination process. Therefore, these measures can only be defined when experiences with the outcome study will be available.

2.2.4. Analyses

a) Research question 1: Effectiveness of the program

Changes in the outcomes of interest between baseline and the three follow up measures in the intervention group and the control group will be compared:

Changes in physical activity behaviour:
- proportions of previously insufficiently active individuals that became regularly active. Results will be given as Relative Risks with 95% confidence intervals.
- proportions of previously regularly active individuals that became insufficiently active. Results will be given as Relative Risks with 95% confidence intervals.
- Distributions of individuals not changing their behaviour (irregularly active / regularly active) and individuals changing their behaviour (irregularly active to regularly active and vice versa). Chi2 statistics for a 2x4 contingency table will be used.
- proportions of subjects that progressed in the TTM-stages.
- changes in caloric expenditure
- changes in minutes of moderate intensity activity per week
- changes in minutes of vigorous intensity activity per week

Changes in knowledge
- changes in proportions knowing the current hepa-recommendations
- changes in the proportions of participants assessing their behaviour correctly as “active enough” or “not active enough”

Change in self-efficacy
- changes in self-efficacy scores

Changes in health related quality of life
- changes in scores on the SF-12 scale

If participants in the two outcome studies do not differ regarding baseline characteristics, data may be pooled for subgroup analyses.

b) Research question 2: Comparison with spontaneous users

The same outcomes listed above will also be assessed for the study group recruited on the website. Outcome differences between the groups in effectiveness studies and the group of the spontaneous users will be compared quantitatively and qualitatively. If user characteristics do not differ, the data can be pooled with the data from the effectiveness studies.

2.3 Bedeutung der geplanten Forschungsarbeit

A large proportion of the Swiss population does not meet the current recommendations for health enhancing physical activities (Martin, 2002). First experiences with the dissemination active-online.ch demonstrate that the program is well accepted and used by a relevant proportion of the Swiss population. The potential for active-online.ch as an intervention to promote physical activity in Switzerland is therefore considerable and the investigation of its effectiveness is essential to estimate its Public Health impact to plan and justify the investment of resources into this one or similar programs in Switzerland.
A study on the effectiveness of active-online.ch to change physical activity behaviour fits well into the first priority (*Promotion of health through physical activity and sports)* of the research sector *Sport and Physical Activity* of the *Research in Swiss Government Departments* 2004 – 2007.
Also on the international level, there are no studies so far assessing the effectiveness of internet-based tailored intervention programs for physical activity or any other health behaviour. Therefore, a study on the effectiveness of active-online.ch will also provide results relevant for the international research and public health community.

2.4 Forschungskompetenz

Brian Martin, M.D., MPH

Brian Martin has worked in health promotion since 1993 and full-time in health promotion through physical activity and sports since 1995. Apart from his work in the development and the implementation of a national strategy for the promotion of physical activity and sports (Martin, 2002; Martin et al, 2000.), his particular scientific interest is in the measurement and in the epidemiology of physical activity (Martin, 2002; Martin, Beeler et al, 2001; Martin et Mäder, 2002; Mäder, Martin et al, 2002) and in the effectiveness of interventions for the promotion of physical activity (Titze, Martin et al., 2001; Martin, Jimmy et Marti, 2001, Martin, 2002).

Martin BW. Perspektiven für bessere Lebensqualität - Was macht das Bundesamt für Sport? In: Colombani PC, Frey WO, Wenk C. Lebensqualität. Beiträge zur Gesundheitsförderung. Manuskripte zur internationalen Fachtag Bewegung - Ernährung - Erholung, Davos, 26.-28.04.2001. Zürich: INW Ernährungsbiologie, ETH Zürich, 2002.

Martin BW, Ulrich U, Bachmann G, Calmonte R, Casabianca A, Casparis C, Mahrer Th, Mathys R, Nützi Ch, Rielle JC, Somaini B. Gesundheitsförderung durch Bewegung und Sport. In: Anonymus. Konzept des Bundesrates für eine Sportpolitk in der Schweiz. Mit Massnahmen zur Förderung von Bewegung und Sport. Werkstattbericht November 2000. Magglingen: Bundesamt für Sport, 2000: 15-33.

Martin BW. Physical activity related attitudes, knowledge and behaviour in the Swiss population: comparison of the HEPA Surveys 2001 and 1999. Schweiz. Schweiz Z Sportmed Sporttraumatol, submitted for publication.

Martin BW, Beeler I, Szucs T, Smala AM, Brügger O, Casparis C, Allenbach R, Raeber PA, Marti B. Economic benefits of the health-enhancing effects of physical activity: first estimates for Switzerland. Scientific position statement of th Swiss Federal Office of Sports, Swiss Federal Office of Public Heal, Swiss Council for Accident Prevention, Swiss National Accident Insurance Organisation (SUVA) , Department of Medical Economics of the Institute of Social and Preventive Medicine and the University Hospital of Zurich and the Network HEPA Switzerland. Schweiz. Schweiz Z Sportmed Sporttraumatol, 2001; 49 (3): 131-133.

Martin BW, Mäder Urs. Körperliches Aktivitätsverhalten in der Schweiz. In: Samitz G, Mensink G (Hrsg). Körperliche Aktivität in Prävention und Therapie. Evidenzbasierter Leitfaden für Klinik und Praxis. München: Marseille Verlag GmbH, 2002.

Mäder U, Martin B, Schutz Y, Marti B. Validation study of short physical activity questionnaires, based on accelerometry. Abstract. In Miilunpalo S, Tulimäki (ed.). International Symposium on

Health-Enhancing Physical Activity (HEPA). Evidence-based Promotion of Physical Activity. Helsinki, Finland, September 1-2, 2002. Book of Abstracts. Tampere: UKK Institute, 2002: 67.

Titze S, Martin BW, Seiler R, Stronegger W, Marti B. Effects of a lifestyle physical activity intervention on stages of change and energy expenditure in sedentary employees. Psychology of Sport and Exercise 2001; 2: 103-116.

Titze S, Martin BW, Seiler R, Marti B. A worksite intervention module encouraging the use of stairs: results and evaluation issues. Soz Präventivmed 2001; 46: 013–019.

Martin BW, Jimmy G, Marti B: Bewegungsförderung bei Inaktiven - eine Herausforderung auch in der Schweiz. Ther Umschau 2001; 58: 196-201.

Martin BW. Bewegung und Gesundheit: Konsequenzen und Möglichkeiten für die hausärztliche Praxis. Primary Care 2002;2:9–11.

Jimmy G, Martin BW. Implementation and effectiveness of a primary care based physical activity counselling scheme. Patient Education and Counselling, 2004, in press.

Martin-Diener E, Thüring N, Melges T and Martin BW. The Stages of Change in three stage concepts and two modes of physical activity: a comparison of stage distributions and practical implications. Health Education Research, 2004, 19: 406-417.

Georg Bauer, M.D., DrPH

Georg F. Bauer has 12 years of experience in development of health promotion programs and in intervention research. During his stay at the School of Public Health in Berkeley he conducted neighbourhood and workplace based intervention studies in collaboration with the Center for Family and Community Health (a CDC Health Promotion and Disease Prevention Research Center) and the Oakland Community Based Public Health Initiative. Since 2001 he chairs the department of health and intervention research at the Institute of Social and Preventive Medicine of the University of Zurich which developed several Internet- and TTM-based expert systems. Currently ongoing studies include a study on the relation between work-related psychosocial factors, subjective health and objective absenteeism data; a study to evaluate effects of worksite health promotion interventions in small- and medium-sized enterprises; Causes and effects of work-life conflicts - a study on the basis of data of the Swiss Houshold Panel.

Bauer G. SME-Vital: Programme for Healthy Enterprises. Abstract (Oral presentation). 4th European Conference on Promoting Workplace Health. Dublin, June 2004

Reuter H, *Bauer G.* Zusammenhänge zwischen selbst- und fremdbeurteilten Arbeitsbedingungen und Gesundheitsindikatoren in einem schweizerischen Detailhandelsunternehmen. Abstract (Oral presentation). Swiss Public Health Conference. Zürich, June 2004

Bauer G et al. European Health Promotion Indicator Development Projekt (EUPHID): zugrundeliegendes Modell und Klassifikationssystem. Abstract (Oral presentation). Swiss Public Health Conference. Zurich, June 2004

Bauer G. Swiss National Program for Comprehensive Workplace Health Promotion in Small and Medium-Sized Enterprise: Development, Implementation & Evaluation. No 2034. Session: Current and emerging priority issues in Workplace Health. Abstract (Oral presentation). World Conference on Health Promotion & Health Education. Melbourne, April 2004

Bauer G. Towards a human resource management oriented approach to worksite health promotion: empirical evidence from the Swiss service sector. No 2039. Session: Making the business case for workplace health promotion. Abstract (Oral presentation). World Conference on Health Promotion & Health Education. Melbourne, April 2004

Thüring N, Martin-Diener E, *Bauer G,* Martin BW. Internet-based randomized study on the effectiveness of the physical activity program www.active-online.ch: a feasibility study. (Final report).

Institute of Social and Preventive Medicine Zurich and Institute of Sport Sciences Magglingen. April 2004

Bauer G, Schmid M, Lehmann K, Kuendig S, Kissling D. Entwicklung und Evaluation eines nationalen Programms für betriebliche Gesundheitsförderung in KMU. Abstract (Oral presentation). Arbeit + Gesundheit in effizienten Arbeitssystemen. 50. Kongress der Gesellschaft für Arbeitswissenschaft. Zürich, März 2004

Hämmig O, *Bauer G.* Ursachen und gesundheitliche Auswirkungen mangelnder Work-Life Balance bei der Schweizer Erwerbsbevölkerung. Abstract (Oral poster). Arbeit + Gesundheit in effizenten Arbeitssystemen. 50. Kongress der Gesellschaft für Arbeitswissenschaft. Zürich, März 2004

Hämmig O, *Bauer G.* Arbeit und Gesundheit im Kanton Zürich. Befragungsergebnisse zu Arbeitsbedingungen, Work-Life Balance und Befindlichkeit. Zürich, März 2004

Schmid M, *Bauer G,* Ammann K, Kuendig S, Kissling D. A Swiss national program for comprehensive workplace health promotion in small- and medium-sized enterprises: development, implementation and evaluation. Abstract (Oral presentation). Annual meeting of the European Public Health Organization. Rome, November 2003

Bauer G, Schmid M. Swiss national program for comprehensive workplace health promotion in small- and medium-sized enterprises. Abstract (Oral presentation). Annual meeting of the American Public Health Organization. San Francisco, November 2003

Bauer G, Davies JK, Pelikan J, Noack H, Broesskamp U, Hill C on behalf of EUHPID Consortium. Advancing a Theoretical Model for Public Health and Health Promotion Indicator Development: Proposal from the EUHPID Consortium. European Journal of Public Health 13;3 Supplement:107-113, 2003

Märki A, *Bauer G,* Conca-Zeller A, Stuck A.E., Gehring T.M. Systematische Bewegungsberatung für ältere Menschen: Ergebnisse einer Längsstudie unter Berücksichtigung des Transtheoretischen Modells Abstract (Oral presentation). Tagung Schweizerische Gesellschaft für Gerontologie und
5. Nationale Gesundheitsförderungskonferenz. St. Gallen, 2003

Märki A, Angst F, *Bauer G,* Stuck A, Gehring G. Systematische Bewegungsberatung für über 65-Jährige im Rahmen einer Pilotstudie: erste Ergebnisse aus dem Projekt Zürcher hausärztliche Bewegungsberatungen für über 65-Jährige. Abstract (Oral Presentation). Jahrestagung der Schweizerischen Gesellschaft für Prävention und Gesundheitswesen. Basel, August 2003

Bauer G. European Health Promotion Model for indicator development. Abstract (Oral presentation).
6th European Conference of the International Union for Health Promotion and Education. Perugia, June 2003

Bauer G, Schmid M. State of potentially stress reducing human resource management in Swiss service companies. Abstract (Oral presentation). Fifth Interdisciplinary Conference on Occupational Stress and Health. Toronto, March 2003

Bauer G. European Health Promotion Indicators Project – a case study. Abstract (Oral presentation).
5th International Union for Health Promotion and Education - European Conference on the Effectiveness and Quality of Health Promotion. London, June 2002

Bauer G. Sample Community health indicators on the neighborhood Level. In: Minkler M, Wallerstein N (eds.) Community based participatory research for health. San Francisco: Jossey-Bass 2002 (p. 438-445)

Bauer G, Schmid M, Zellweger U, Krueger H. Betriebliches Gesundheitsmanagment 2001 – Entwicklungsstand in Schweizer Dienstleistungsunternehmen (“Integration of health management and quality management in the Swiss service industry”). Schlussbericht Projekt No 32-56055.98 des Schweizer Nationalfonds. Institut für Sozial- und Präventivmedizin Zürich. Zürich, Februar 2002

Bauer G, Schmid M, Krueger H. Integration of worksite health promotion and quality management: a survey in the Swiss service industry. Abstract (Oral presentation). Annual Conference of the European Public Health Association (EUPHA). Brüssel, December 2001

2.5 Zeitplan des Projekts

	2005						2006					
Preparations; recruitment of org. units	■	■										
Programming of website		■	■									
Data collection I: baseline				■								
Data collection II: 1 month follow-up					■							
Data collection III: 6 month follow-up							■					
Data collection IV: 12 month follow-up										■		
Analysis I					■	■						
Analysis II							■	■	■			
Analysis III										■	■	
Intermediate report						■						
Final report, Publications										■	■	■

3. Vorhandene Ressourcen

Was steht dem Gesuchsteller zur Zeit ohne die in diesem Gesuch beantragten Mittel zur Bearbeitung des vorliegenden Projekts zur Verfügung?

3.1 Personal

Project coordination and supervision by the person responsible for the development of the program active-online.ch and the feasibility study (Eva Martin-Diener)
CHF 15'000.-

Project supervision by the Institute for Social and Preventive Medicine, University of Zürich CHF 10'000.-

Analyses of accelerometer data by the Institute of Sports Sciences, Federal Office of Sports, Magglingen CHF 10'000.-

Programming of the online version of the Physical Activity Frequency Questionnaire (Bernstein et al., 1998) by the Institute of Sports Sciences, Federal Office of Sports, Magglingen CHF 10'000.-

3.2 Vorhandene Apparate

Hosting, monitoring and technical service of the program active-online.ch
CHF 40'000.-

3.3 Kredite für neue Apparate, Verbrauchsmaterial, Reisen und weitere Ausgaben

----

3.4 Infrastruktur (Räumlichkeiten, Werkstätten, Rechenzentren, Bibliotheken, usw.)

Fully equipped office including computers for analyses

3. Von anderer Seite beantragte Mittel

4.1 Wurden für die Unterstützung dieses Projekts bei anderen Geldgebern Mittel beantragt oder ist beabsichtigt, dies zu tun?

☐ ja X nein

4.2 Wenn ja, um welche Institution/Institutionen handelt es sich?

Name:

Adresse:

Telefon:

Fax:

e-mail:

4.3 Welchen Umfang machen die beantragten Mittel aus?

Fr.

4.4 Bis wann ist ein Entscheid zu erwarten?

4. Finanzieller Bedarf

Welche Mittel werden während der Projektlaufzeit ausserdem benötigt?

Please notice that for this study we will need computer software and advertisements, not technical apparatus

5.1 Material von bleibendem Wert

(z.B. Apparate und Instrumente, die im Handel erhältlich sind)

Programming and graphic design of the study website CHF 20'000.-

5.2 Bauelemente, Einbaugeräte, Bestandteile

(z.B. Bestandteile für den Bau oder Ausbau von Apparaturen und Systemen)

5.3 Verbrauchsmaterial

Advertisements for the recruitment of participants CHF 45'000.-

5.4 Unterhalt von Apparaturen

Hosting of the study website (provider) CHF 4000.-

5.5 Reisen, Kongresse, Feldspesen, usw.

Congresses CHF 4000.-
Travel expenses, literature, reserve CHF 4000.-

6. Personalbedarf

6.1 Saläre

1) Name: **NN** Vorname:
Nationalität: Zivilstand:
AHV-Nr.: Anzahl Kinder:
Akad. Grad oder Diplom: seit (Monat/Jahr):
Doktorand: ☐ ja, seit: ☐ nein
Funktion: % Arbeitszeit: 70%
Salär 1. Jahr: CHF 47'000.- Salär 2. Jahr: CHF 47'000.-

The person who will conduct this study is not yet assigned. A highly qualified researcher will be needed for this complex study design and demanding data analyses.

2) Name: Vorname:
Nationalität: Zivilstand:
AHV-Nr.: Anzahl Kinder:
Akad. Grad oder Diplom: seit (Monat/Jahr):
Doktorand: ☐ ja, seit: ☐ nein
Funktion: % Arbeitszeit:
Salär 1. Jahr: Salär 2. Jahr:

3) Name: Vorname:
Nationalität: Zivilstand:
AHV-Nr.: Anzahl Kinder:
Akad. Grad oder Diplom: seit (Monat/Jahr):
Doktorand: ☐ ja, seit: ☐ nein
Funktion: % Arbeitszeit:
Salär 1. Jahr: Salär 2. Jahr:

6.2 Sozialabgaben (Arbeitgeberbeitrag an AHV, IV, usw.)

CHF 15’980.- (Arbeitgeber- plus Arbeitnehmerbeiträge: 17%)

6.3 Versicherungen

included in 6.2

7. <u>Rekapitulation der gewünschten Mittel</u>

5.1	Fr. 20'000.-		
5.2			
5.3	Fr. 45'000.-		
5.4	Fr. 4'000.-		
5.5	Fr. 8'000.-	Fr.	77'000.-
6.1	Fr. 94'000.-		
6.2	Fr. 15'980.-		
6.3	Fr. ___	Fr.	109'980.-
Total		Fr.	186'980.-

Ort und Datum:

Magglingen, 14.9.2004

Unterschrift des Hauptgesuchstellers/
der Hauptgesuchstellerin:

....

Ort und Datum:

Zürich, 14.9.2004

Unterschrift des Mitgesuchstellers/
der Mitgesuchstellerin:

....

<u>Beilagen</u>:

X Lebenslauf des Hauptgesuchstellers/der Hauptgesuchstellerin
X Lebenslauf des Mitgesuchstellers/der Mitgesuchstellerin
X Liste einschlägiger Publikationen

<u>Anhang1</u>: Nennung von 3 Experten zur Thematik des Gesuchs
